# Neutron Scattering Studies of the Interplay of Amyloid β Peptide(1–40) and An Anionic Lipid 1,2-dimyristoyl-sn-glycero-3-phosphoglycerol

**DOI:** 10.1038/srep30983

**Published:** 2016-08-09

**Authors:** Durgesh K. Rai, Veerendra K. Sharma, Divina Anunciado, Hugh O’Neill, Eugene Mamontov, Volker Urban, William T. Heller, Shuo Qian

**Affiliations:** 1 Biology and Soft Matter Division, Oak Ridge National Laboratory, Oak Ridge, TN 37831, USA; 2 Chemical and Engineering Materials Division, Oak Ridge National Laboratory, Oak Ridge, TN 37831, USA

## Abstract

The interaction between lipid bilayers and Amyloid β peptide (Aβ) plays a critical role in proliferation of Alzheimer’s disease (AD). AD is expected to affect one in every 85 humans by 2050, and therefore, deciphering the interplay of Aβ and lipid bilayers at the molecular level is of profound importance. In this work, we applied an array of neutron scattering methods to study the structure and dynamics of Aβ(1–40) interacting 1,2-dimyristoyl-sn-glycero-3-phosphoglycerol (DMPG) bilayers. In the structural investigations of lipid bilayer’s response to Aβ binding, Small Angle Neutron Scattering and Neutron Membrane Diffraction revealed that the Aβ anchors firmly to the highly charged DMPG bilayers in the interfacial region between water and hydrocarbon chain, and it doesn’t penetrate deeply into the bilayer. This association mode is substantiated by the dynamics studies with high resolution Quasi-Elastic Neutron Scattering experiments, showing that the addition of Aβ mainly affects the slower lateral motion of lipid molecules, especially in the fluid phase, but not the faster internal motion. The results revealed that Aβ associates with the highly charged membrane in surface with limited impact on the structure, but the altered membrane dynamics could have more influence on other membrane processes.

Amyloid β peptide (Aβ) is the constituent of the amyloid fibril plaques found in Alzheimer’s disease patients. Alzheimer’s disease (AD) is the most common form of dementia and is predicted to affect 1 in 85 people around the world by 2050^1^. Aβ is typically composed of 36–43 amino acids that are resulted from cleavage by secretases from a transmembrane glycoprotein amyloid precursor protein (APP) near membranes. While the actual physiological function of Aβ is yet to be established, it is regarded as an important factor in the development of AD, given the fact that the Aβ accumulation as fibril plaques in the neuropil is one of the pathological hallmarks of AD^2^,^3^,^4^,^5^,^6^,^7^,^8^,^9^. The insoluble proteinaceous Aβ plaques associated with brain mass loss are primarily composed of Aβ fibril aggregates. However, the toxicity of Aβ is yet to be determined since Aβ is present in a normal person’s brain without known harmful effect. Besides, there is no consistent observation that the total Aβ concentration is elevated in AD brain^10^,^11^. In previous studies, Aβ is found at very low concentrations and has an unordered structure in normal biological fluids at physiological pH^12^,^13^,^14^,^15^. It exhibits profound secondary structure changes among the different transition pathways between random coils, α-helix, and β-sheet under different conditions^16^,^17^. It tends to self-aggregate into soluble oligomers and eventually forms amyloid fibrils^12^,^18^,^19^,^20^,^21^. Recent consensus is that the lower-molecule weight oligomers of Aβ, many of which are soluble in solution and readily bind to neuron cell membranes, cause greater harm to the membrane^22^,^23^,^24^,^25^. Moreover, the cleavage site of APP is adjacent to membrane bilayer^26^,^27^,^28^. Therefore the plasma membrane has been suggested as the actual target for the cytotoxicity of Aβ and some studies have shown that Aβ can cause extensive damage on cell membrane^22^,^29^,^30^,^31^. However, the cause of the damage is unclear owing to the lack of molecular details on the interaction between Aβ and lipid bilayers in establishing Aβ cytotoxicity^32^,^33^. With recent development in neutron sources and instrumentations, neutron scattering methodologies afford us to examine such system in great details with the advantages of neutron contrast variation in scattering^34^ and fine energy resolution at different length scales in neutron spectrometer^35^. In the present work, we demonstrated that the neutron techniques, including Small Angle Neutron Scattering (SANS), Neutron Membrane Diffraction (NMD), and Quasi-Elastic Neutron Scattering (QENS), for the first time to our knowledge, reveal the underlying molecular interactions between Aβ and model membrane systems composed of a negatively charged lipid, 1,2-dimyristoyl-sn-glycero-3-phosphoglycerol (DMPG).

While the majority of the APP cleavage product is composed of two Aβ peptides with different lengths, Aβ(1–40) and Aβ(1–42), we used the more predominant Aβ(1–40) in this studies. The two extra hydrophobic residues near the c-terminal of Aβ(1–42) makes it more hydrophobic and of much stronger amyloidgenicity with rather faster kinetics. In contrast, Aβ(1–40) possess a more defined structure over long period of experimental time required by neutron techniques employed in this studies. With the continuing developments of the instrumentations, the same methodologies can be applied to resolve Aβ-membrane systems with faster kinetics. Hereafter, Aβ(1–40) in the text is referred as Aβ.

The model membrane systems used in our studies consist of anionic lipid, which have been found to increase Aβ binding to bilayers previously^28^,^36^,^30^. But this doesn’t rule out that Aβ has considerable interaction with neutral lipids. The net charge carried by Aβ varies with pH conditions. The section of residues 1–28, which is suspected as non-membrane inserting extracellular part, has 6 acidic amino acids and 6 basic amino acids at neutral pH. The other part of the peptide is uncharged and is of significantly hydrophobic. With charged lipids, the surface of lipid membrane is of intricate nature with transitional polarity, and the electrostatic interactions between lipid and peptide could result in substantial reaction enthalpies^37^,^38^. This has implicated that lipid bilayer not only provides a suitable interface favoring the assembling of Aβ into toxic species, but also becomes a possible target for Aβ cytotoxicity. To exploit the stronger interaction between charged lipids and Aβ, we used anionic DMPG as the sole lipid composition. Two model membrane systems were used: unilamellar vesicle (ULV) in solution provides a system close to physiologically relevant condition to observe the lipid bilayer change and Aβ transformation by SANS and QENS, while the substrate-supported multilamellar bilayer provides a more ordered system to resolve details of Aβ in bilayers by NMD. The combined results showed that Aβ associates with the surface of lipid bilayer, as recent studies have pointed toward an affirmative interaction of lipids membranes with Aβ^28^,^36^, but with limited ability to insert deeply into bilayer in the presence of highly charged lipid. However, the association significantly modifies the lateral dynamics of the lipid. The neutron techniques revealed that even the membrane bilayer structure is relatively intact; Aβ binding alters the membrane bilayer property that could affect other cellular function based on membrane. Also the “surface binding” nature of the interaction induced by charged lipid could enable Aβ to assemble into oligomers or aggregate on the extended surface provided by cell membranes.

## Results

### Interplay of Aβ and DMPG bilayer in ULVs

To determine the optimum concentration at which a significant portion of Aβ binds and interacts with DMPG bilayer in solution, we performed a series of CD experiments with different concentrations of Aβ added to ULVs sample solutions. All scans were performed at 30 °C after equilibrating the samples for ~2 h after titrating different amount of Aβ into 3 mM ULVs solution, with the effective peptide-to-lipid molar ratio of 1/150, 1/100, 1/50 and 1/30 (Figure S1(a)). The quantitative analysis of CD data in 185–240 nm data range^39^, shows that in the presence of ULVs at any concentration, Aβ conformations are distinct from the control Aβ without presence of lipids Table S1. Aβ in control solution is largely in random coil conformation, with minor presence of β-sheet/turns and α-helices. After addition of DMPG, β-sheet/turns increases from 16% to about ~33 ± 2% then remains somewhat constant throughout the series. Thereafter, a smaller shift of random conformations to α-helices takes place as the peptide-to-lipid molar ratio decreases. The conformational changes, even at low peptide-to-lipid ratio, indicate significant amount of Aβ binding to DMPG ULV bilayer. To maximize the peptide lipid interaction and reflect physiological conditions with abundance of Aβ in plasma membranes, we use peptide-to-lipid molar ratio of 1/30 for most of our other experiment with ULV.

The effect of DMPG ULVs on Aβ was also evident by using FTIR, where three peaks corresponding to structural changes in Aβ were investigated, as shown in Figure S1(b)^40^,^41^. The peak at 1635 nm, which corresponds to the β-sheet, undergoes transitions from a maximum at 1635 nm in the absence of lipid to a minimum at the same wavelength, similar to previous studies^40^,^41^,^42^. The peak intensity at 1652 nm, which corresponds to the presence of random coil, decreases for the DMPG/Aβ sample in comparison with the control Aβ sample^43^. Also the shoulder at 1683.3 nm with respect to the peak at 1684.5 nm decreases in the presence of DMPG, which also suggest formation of β-sheet^44^,^45^. Both experiments indicate that the Aβ does change secondary structure in the presence of DMPG ULVs with respect to the control Aβ under similar conditions of concentration and temperature, showing that DMPG and Aβ positively interact with each other. However, it cannot provide an answer to the location of Aβ with respect to lipid bilayer.

To get the initial structural parameters such as ULVs sizes and polydispersity for SANS data fitting, DLS was performed using 20 independent acquisitions over 4 seconds each. The diffusion coefficient *D*_*0*_, derived from DLS data, was reduced by the addition of Aβ (in 1/30 P/L ratio) from 4.6 ± 0.1(x10^−8^ *cm*^2^*/s*) to 4.3 ± 0.2(x10^−8^ *cm*^*2*^*/s*) with slight increases in the overall effective hydrodynamic radius, *R*_*h*_, from ~48.6 ± 1.4 nm to 50.5 ± 1.8 nm. From our DLS measurements, we also confirmed that the vesicles are intact under the presence of Aβ and the addition of Aβ does not significantly change the size distribution and polydispersity of the ULVs.

**Figure 1:**
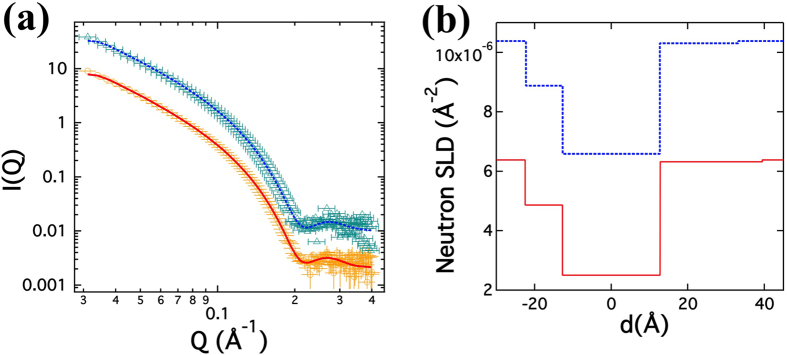
(**a**) SANS on DMPG (Orange circles) and DMPG/Aβ (Cyan triangles) ULVs at 30 °C at a concentration of 20 mg/ml under 100% D_2_O with respective Poly Core 3-Shell fits in red and blue lines respectively and (**b**) corresponding fitted neutron scattering length density profiles in red lines and blue dashes. The data for DMPG/Aβ has been offset for visual clarification.

SANS experiments were performed on DMPG ULV samples of 20 mg/ml (2% w/w) with (peptide-to-lipid ratio = 1/30) and without Aβ at 30 °C where membranes are in fluid phase. The reduced scattering *I-Q* plots are shown in Fig. 1(a). As a neutron technique sensitive to size and scattering length density change, SANS can be used to elucidate the structures at different locations. The core-shell model fit results of SANS data are shown in Fig. 1(b) and numerically presented in Table 1. The fits for the headgroup region, in particular the outer headgroup region, reveal a higher SLD due to permeation of D_2_O. While the presence of D_2_O in the headgroup region is anticipated, the values of significantly high SLD of outer headgroup in particular, seem to suggest that the charged DMPG headgroup enhances the presence of water in vicinity^46^. The results concur with previous observations about hygroscopic nature of anionic lipids^47^. The thickness of inner headgroup and the hydrocarbon chain region remain similar at about 10 Å and 25 Å, respectively, for both samples. The outer headgroup thickness shows a decrease upon addition of Aβ. On the other hand, the SLDs from the fits are found to be close to the bulk D_2_O outside the vesicle, probably owing to hygroscopicity of PG or more disordered outer layer induced by Aβ, thus contributing little to the overall scattering intensity. Therefore, the fitted parameters as well as the rather insignificant variations therein indicate that the impact of Aβ is limited to the internal bilayer structure.

**Figure 2:**
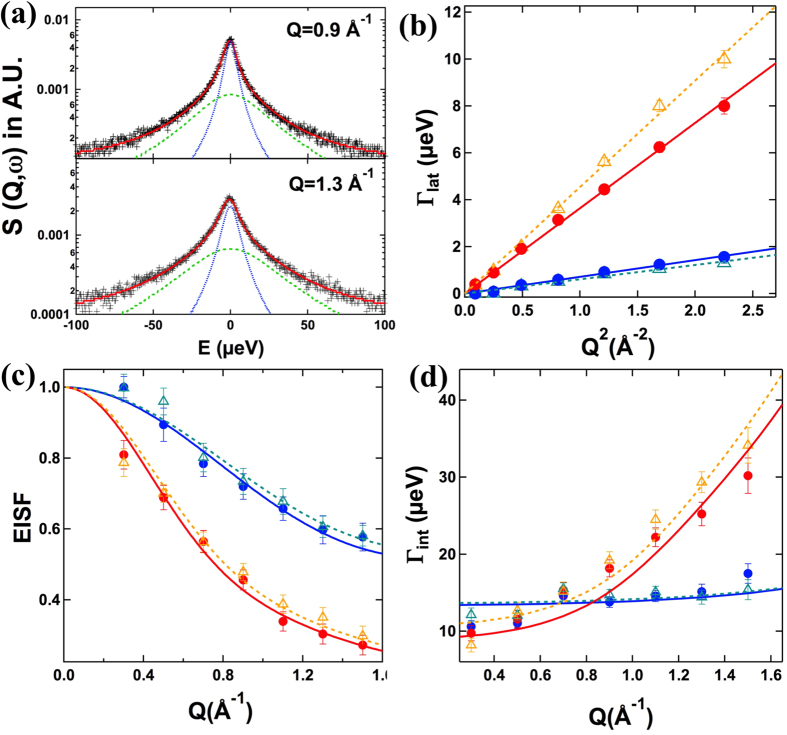
(**a**) Fitted S_vesicles_(Q, ω)(red) for 5 wt% DMPG/Aβ vesicles at 37 °C (black plus sign) assuming the model scattering function given by Eq. (5). The blue and the green dash lines correspond to lateral and internal motions respectively. Variation of (**b**) half width at half maximum (HWHM) for lateral motion, Γ_lat,_ vs. Q^2^ with corresponding fits with Fick’s law of diffusion (**c**) EISF vs. Q along with the (d) HWHM for internal motion, Γ_int_ vs. Q; for DMPG ULVs in the solid gel (7 °C in solid blue circles) and fluid (37 °C in solid red circles) phase and DMPG/Aβ ULVs in the solid gel (7 °C in cyan triangles) and fluid (37 °C in orange triangles) phase.

A closer look at the results from DLS and SANS suggest that the overall size of the DMPG ULVs increases after the binding of Aβ even as the actual bilayer thickness decreases, possibly under the influence of Aβ on its surface, similar to other membrane binding peptides seen before^34^,^48^. Thereafter, high resolution QENS was used to understand the underlying dynamics of Aβ-lipid interaction. QENS technique is sensitive to motions in a wide range of time scale from pico- to nano-second and on length scales from Ångstroms to tens of nanometers. The QENS data analysis (Fig. 2(a)) separately shows two different kinds of motion, namely lateral motion of whole lipid molecules within the monolayer and internal motion of lipid molecule, by the scattering law described in equation (5) (Fig. 2(a)). The effect of Aβ on the lateral motion of the lipid molecules is compared using the variations in the half width at half maximum (HWHM) at two different temperatures, 7 °C and 37 °C, respectively, as shown in Fig. 2(b). It is evident that the lateral motion of lipid molecules follows continuous diffusion model (*Γ*_lat_ = *D*_lat_*Q*^2^) at both temperatures in presence and absence of Aβ. Lateral diffusion coefficient (*D*_*lat*_) can be obtained from the slope between *Γ*_*lat*_ and *Q*^*2*^ and is given in Table 2. At 7 °C, the presence of Aβ does not appreciably affect the lateral motion of DMPG molecules, as little difference in variation of HWHM is observed in DMPG vesicles in presence and absence of Aβ. This is understandable on the grounds that in the gel phase, the chain is less prone to any interaction induced by other molecules^49^. At 37 °C, in the fluid phase of DMPG, the effect of Aβ is evident by apparent difference in variation of HWHM, where the presence of Aβ leads to enhancement in lateral diffusion coefficient. The addition of Aβ in the fluid phase leads to effective thinning of the bilayer, as observed from SANS analysis (Table 1), which could result in more area per lipid molecules and an increase in the lateral diffusion coefficient.

The internal motions of lipid can be quantified using elastic incoherent structure factor EISF, shown in Fig. 2(c) for DMPG vesicles in presence and absence of Aβ. Variation of HWHMs corresponding to internal motion for both vesicles solutions are shown in Fig. 2(d). Following the method developed recently by Sharma *et al.*^35^ on describing the internal motion of DMPC (1,2-dimyristoyl-sn-glycero-3-phosphocholine) molecules in vesicles, the gel phase and the fluid phase were treated differently. In gel phase, the fractional uniaxial rotational model (equation (S5–S6)) is employed to describe data for DMPG vesicles in the presence and the absence of Aβ at 7 °C^50^. DMPG molecules were found to exhibit essentially the same internal dynamics: 55 ± 3% of hydrogen was found to undergo uniaxial rotation on a circle of 1.6 ± 0.1 Å in DMPG ULVs only sample; the fraction of mobile hydrogen and radius of circle are found to be 52 ± 3% and 1.5 ± 0.1 Å respectively, upon addition of Aβ. Concurrently, the HWHM of internal motion (*Γ*_*int*_) is found to be independent of *Q* for both the systems. The value of the rotational diffusion constant *D*_*r*_ is obtained by the least-square fitting of the data assuming a uniaxial rotational diffusion model (equations (S1–S6)). The rotation radius *r* obtained from the EISF *Q*-dependence (Fig. 2(c)) is used as a fixed parameter in the fitting procedure. The solid line at 7 °C in Fig. 2(c) shows that the uniaxial rotation model describes the data well, and the rotational diffusion coefficient for DMPG molecules in the absence and presence of Aβ is found to be similar, with a value of 13 ± 1 *μeV*.

Above phase transition temperature of DMPG, the fluid phase acyl chains are disordered and undergo localized transitional diffusion. The diffusional motion of hydrogen atoms along the chain are within spheres which increase linearly in size from near the headgroup to the end of the tail. Therefore, modified Volino and Danoux model (see SI for details) is used to describe the scattering law^51^. Equation (S10) is used to describe EISF for DMPG vesicles in absence and presence of Aβ at 37 °C, and is found to describe this parameter well. The HWHM of the internal motion at 37 °C is computed numerically for given values of *R*_*min*_, *R*_*max*_, *D*_*min*_ and *D*_*max*_ using Equations (S7–S9). The least-squares fitting method is used to describe the observed HWHM corresponding to the internal motion with *D*_*min*_ and *D*_*max*_ as parameters, while the values of *R*_*min*_ and *R*_*max*_ are kept fixed as obtained from the fit of the EISF (Fig. 2(c)). For DMPG vesicles, the localized motions of the hydrogen atoms along the acyl chains are confined to spheres of radii varying between 0.02 to 4.9 ± 0.3 Å, with corresponding diffusion coefficient of 1.5 Å and 61 ± 5 × 10^−7^ cm^2^/s, respectively. Small values of R_min_ and D_min_ should be taken with reservation; they merely reflect the negligible movement of the hydrogens in the first carbon position held by the headgroup. It is found that addition of Aβ does not influence much the internal motion as for DMPG/Aβ, R_max_ and D_max_ are found to be *4*.*6* ± *0*.*2 *Å and *64* ± *5* × 10^−7^ cm^2^/s, respectively.

It is clear that the addition of Aβ to DMPG ULVs mainly affects the lateral motion of lipid molecules. This is in line with our recent observations of the effect of melittin on the nanoscopic dynamics of the membrane^52^. Melittin has been found to alter mainly the lateral motion of the lipids^52^. This could be explained on the grounds that the lateral motion involves whole lipid diffusion along the monolayer, unlike the internal motion, which is localized in character. Therefore, lateral motion is majorly affected when additive is located at the surface of the membrane. On the other hand, when additive resides deep in the hydrophobic membrane core, like α-tocopherol, both lateral as well as internal motions are found to be affected^53^. Observed results suggest that the Aβ is integrating with the lipid bilayer near the surface of the membrane.

### Interplay of Aβ and DMPG bilayer in multilamellar sample

**Figure 3:**
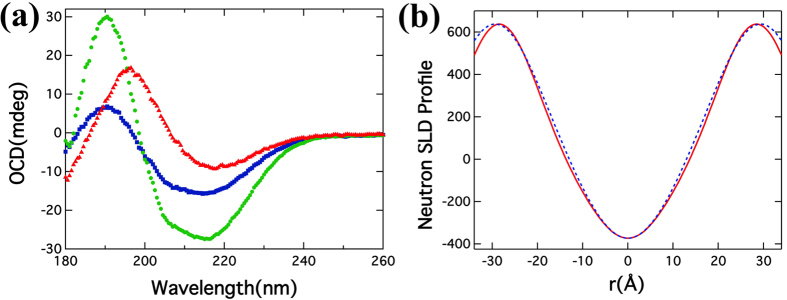
(**a**) OCD from multilayer DMPG/Aβ films where red, green and blue lines indicate as dry cast, equilibrated and wet films respectively and (**b**) Neutron SLD profile using bilayer films of DMPG and DMPG/Aβ at relative humidity of 90% under D_2_O environment, where the red and blue line represent the pure DMPG and DMPG/Aβ NSLDs respectively.

In multilamellar film samples, the lipid molecules self-assemble into oriented bilayers parallel to the surface of the substrate. The well-aligned lipid bilayer samples can be studied by OCD and NMD to provide more details on the interactions between peptide and lipid bilayers. OCD was performed on bilayer films on quartz substrate to investigate orientation of Aβ in bilayers, shown in Fig. 3(a). The Aβ in the multilamellar membrane film (peptide-to-lipid molar ratio of 1:30), is found to be mostly in oriented β-sheet configuration on the surface of the bilayer, similar to that of another β-peptide RTD-1, with the backbone is parallel to the plane of the bilayer^54^,^55^. Thereafter, the film was equilibrated under saturated H_2_O environment at ~37 °C for ~3 hours, after which the OCD was measured as equilibrated samples with RH ~97%. As shown in green in Fig. 3(a), the OCD changes significantly as compared to the dry cast sample, and the presence of α-helices becomes evident in the spectrum. The spectrum resembles a surface state of helices in which the peptide is oriented parallel to the plane of the bilayer^54^. The blue shift of one of the minima from 222 nm to around 215 nm indicates the presence of β-sheet among the peptide. Later, a water droplet ~3 μL was added to the film to over-saturate the sample. With the excessive water, we expected the spacing between the swollen bilayers to increase and become similar to vesicle bilayers in solution, thus the space constraint on the peptide was reduced to allow more flexible configuration in the bilayers. However, the OCD (blue in Fig. 3(a)) remains more or less the same as in the equilibrated state, with only the signal being proportionally reduced due to possible loss of sample on the substrate with excessive water. This showed the peptide and bilayer have already been at equilibrium in the sample at ~97% RH, and the excessive water didn’t change peptide-bilayer interaction. As the peptide on the surface of the membrane is either (mostly) as β-sheets or α-helices parallel to the plane of the bilayer, we conclude that the peptide does not inserts into bilayers significantly.

The NMD data was obtained at a RH of 90%. The NMD data was analyzed to obtain the neutron SLD profile as shown in Fig. 3(b). The *d*-spacing, determined from the peak positions, slightly increased from 56.9 ± 0.2 Å (DMPG only) to 58.7 ± 0.4 Å (Aβ/DMPG = 1/30). The *d*-spacings are not a measure of the membrane thickness since the peaks are representatives of filled D_2_O in between the two headgroups, and the D_2_O has the highest neutron SLD. As a higher spatial resolution technique compare to SANS, the presence of peptide residing inside the hydrocarbon chain is detectable in the SLD profile. No noteworthy changes were observed in central hydrocarbon chain region. At the high concentration of peptide (peptide/lipid = 1/30) used for present study, the bilayer profile would have changed significantly upon insertion of peptide therefore our results suggest the presence of Aβ on the membrane surface. Also the bilayer is symmetric, compared to the profile from ULV solution scattering. This is due to the sample geometry where the multilamellar sample of peptide and lipid are uniform, and there is no distinction between inner and outer leaflet in multilamellar bilayer samples.

## Discussion

Our results indicate that Aβ behaves in a consistent way when interacting with lipid in both unilamellar vesicle bilayers and multilamellar bilayers. Aβ associates in between the interfaces of water-headgroup and headgroup-hydrocarbon chain of lipid bilayer, and does not get inserted into the chain region. There is no penetration into the bilayer from our results of the structural studies. Correspondingly, the QENS results show that Aβ only affects the lateral motion of lipid, but not the fast internal motion. Similar interfacial binding was observed in previous studies with other amyloidogenic peptides or proteins^56^,^57^. This state extends to the highly charged PG lipid bilayers under present study. As a result, the ULVs with relatively high concentration of peptide were intact during the duration of our experiments over a few days.

The interfacial association of Aβ in bilayer signifies the role of membrane in the advancement of Aβ toxicity. The lipid bilayer, being the major structural component of membrane, is able to promote the conformational changes of Aβ. The results from the CD, FTIR experiment with ULVs and OCD experiment with multilamellar bilayers have shown changes in Aβ secondary structures induced by lipid bilayer. The peptide itself, which is predominantly in random-coil conformation, makes a transition to a mixture of β-sheet/turn, random coil and α-helices in the presence of lipid bilayer, consistent with reported studies. The membrane-enabled folding is a critical precondition for Aβ to assemble into various forms such as oligomers and protofibril.

Once bound with the lipid, we have found that Aβ activity is localized near the headgroup region of negatively charged lipid. One possible reason is that the negative charges carried by PG in the bilayer promote accumulation of protons in the vicinity of headgroup in a pH neutral solution. This effectively decreases interfacial pH and changes the Aβ conformation to make it more aggregation-prone, and further modulates the nucleation propensity. More importantly, the strong electrostatic effect exerted by the PG headgroup not only promotes Aβ binding to the lipid bilayer, but may also prevent the further insertion and aggregation due to different charges Aβ carries along with it, as observed in our model single lipid composition bilayers. Both electrostatic and hydrophobic effects are involved in the typical Aβ-membrane interaction. The α-helical conformation of Aβ usually indicates the peptide being inserted into the hydrophobic lipid chain region^58^,^59^. Our results have shown only 10% of secondary structure in α-helix with ~35% as β-sheets conformation, even at very high concentration of peptide, thus peptide was restricted within the interfacial region with an exception of OCD on multilamellar sample. Even in OCD, while a significant presence of α-helix is observed, the orientation of the peptide is mostly in a surface state parallel to the bilayer, with a small fraction perpendicularly inserted under higher humidity and equilibrium conditions^60^. The presence of higher α-helix presence in OCD can be explained by the fact that the relatively drier conditions forced Aβ to reside beyond the interfacial regions with more hydrophobic interactions, but no deep insertion was observed as the bilayer profiles from NMD show no peptide presence in the bilayer chain regions. The strong electrostatic interaction anchors Aβ firmly within the polar interfacial area, and impedes the hydrophobic interaction. At the same time, this strong electrostatic interaction slows down aggregation in an unexpected way. In unilamellar vesicle bilayers, the charge can build up high concentration of Aβ on the membrane surface locally, which is suggested to be disruptive to the membrane with propensity to aggregate and fibrillate. But in our studies, we have found that the ULVs survived high concentration of Aβ for days. The single charged lipid composition might limit Aβ ability to further aggregate or nucleate as the peptide is firmly confined within headgroup with lipids surrounded. Other studies have found a certain ratio of charged lipid in membrane to maximize amyloidgenic activities^61^. Therefore, the balance of charged and non-charged lipids is very important in Aβ-membrane interaction. While the binding and association of Aβ is interfacial, QENS has detected significant modification on the membrane lateral motion, which is important for many membrane functions including signaling and energy transduction pathways. The altered membrane dynamics could have complications for cellular functions associated with interactions of proteins and other molecules inhabiting inside the membrane.

In summary, we have applied various neutron scattering techniques to understand the impact of Aβ on the structure and dynamics of a complex Aβ-membrane system. SANS provides lipid bilayer structure of vesicle in a physiological relevant solution condition while NMD can provide higher resolution bilayer structure in multilamellar form. QENS results indicate that membrane dynamics changes due to incorporation of Aβ. With other readily available biophysical techniques, the neutron scattering techniques provide a unique perspective on the structure and dynamics of such complex system.

## Materials and Methods

### Materials

DMPG was purchased from Avanti Polar Lipids, Inc. (Alabaster, AL.). D_2_O (99.9% atomic D) was purchased from Cambridge Isotope Laboratories (Andover, MA.). Aβ are synthesized by Biopeptide Co. Inc. (San Diego, CA). All materials were used as delivered.

### Peptide Stock Solution Preparation

The stock solution for peptide was prepared by mixing peptide in 50% acetonitrile and freezing at −80 °C before lyophilizing overnight. The lyophilized sample was then resuspended in 2.5 mM NaOH and sonicated under ice mixed water for ~10 minutes^62^. The solution was then frozen at −80 °C for 60 minutes before lyophilizing. Finally the Aβ was titrated in 10 mM NaHPO_4_ (pH ~7.4) as peptide stock and concentration measured with UV-Vis^62^.

### Unilamellar Vesicle (ULV) Sample Preparation

The stock solution for lipid was prepared by dissolving DMPG in chloroform and then drying under vacuum at ~45 °C for 2 h. The lipid was then mixed with D_2_O in appropriate amount and vortex mixed for ~10 minutes. The solution then went through four freeze-thaw cycles by alternately placing the suspension in a warm water bath (50 °C) and in a freezer (−80 °C) each for ~30 minutes. The suspension was then vortexed before extruding through a mini-extruder from Avanti Polar Lipids (Alabaster, AL.) fitted with Whatman^®^ Nuclepore™ membranes with an average pore diameter of 100 nm and equilibrated at ~40 °C. The extrusion was performed in sets of 10. Dynamic light scattering (DLS) (Wyatt Technology Corp., Santa Barbara, CA) was used to determine hydrodynamic size distribution and dynamic parameters. DLS clearly indicated uniform vesicle size for both DMPG and DMPG/Aβ vesicles, respectively.

### Multilamellar Film Sample Preparation

The ULV samples prepared using the above procedure were deposited onto 19 *mm* × 19 *mm* × 0.5 *mm* thick quartz substrate and left for 3–4 h for the water vapors to slowly evaporate under room temperature and atmosphere^63^,^64^,^65^. The residual water content in samples was further reduced by vacuum for about 2 h. Thereafter the samples were rehydrated either in sealed jar with D_2_O for NMD or with H_2_O for OCD experiments, for overnight at ~37 °C. The film looked uniform upon visual inspection and was used within 2 days of preparation for NMD and OCD measurement. The total lipid amount for each sample on the substrate for NMD experiment is ~5 mg, while the lipid amount was reduced to ~500 μg to increase the transparence to UV light in OCD measurement.

### NMD Data Collection and Reduction

NMD data was collected at the Bio-SANS instrument at the High-Flux Isotope Reactor in Oak Ridge National Laboratory using a neutron wavelength of 6 Å^66^. The wavelength spread, *Δλ*/*λ* was set to 0.15 by a neutron velocity selector. A sample-to-detector distance (SDD) of 1.13 m with rocking scans over an angle of 4° was employed to acquire NMD data. The data was reduced using in-house developed routines implemented MATLAB software (MathWorks Inc., Natick, MA). The diffraction peak intensity was obtained by integrating the peak from a reduced 2D *Q*_*xy*_ map, normalized to incident beam monitor counts, corrected for detector dark current, pixel sensitivity and background correction. The diffraction amplitudes obtained from the integrated diffraction peak intensity from the reduced 2D detector images were corrected for the Lorentz factor, the absorption correction factor and the geometry correction factor^63^,^67^,^68^. In total, three orders of peaks were used to calculate the scattering length density profiles (Figure S2).

The phases for amplitudes were determined by fitting the evaluated amplitudes from different relative humidity using the swelling method^69^ (Figure S3). Thereafter the SLD profiles were calculated and normalized to obtain the scattering length density profile for the bilayer^67^,^68^.

### SANS Data Collection and Reduction

Cylindrical quartz cells (Hellma, Germany) with 1 mm path lengths were used to collect SANS data on ULVs at the Bio-SANS at the High-Flux Isotope Reactor or EQ-SANS instrument of the Spallation Neutron Source, both located at Oak Ridge National Laboratory. For the Bio-SANS experiment, wavelength of neutron was set to 6 Å with the wavelength spread, *Δλ*/*λ* ~ 0.15 by a neutron velocity selector. The EQ-SANS measurements employed a sample-to-detector distance of 4 m and the instrument was used in 30 Hz mode with a minimum wavelength setting of 2.5 Å. For both instruments, configurations were used to provide an effective *q*-range of ~0.03 Å^−1^ to 0.45 Å^−1^. All SANS measurements were performed at 30 °C with ULVs concentration of 2% w/w, well above the phase transition temperature of DMPG (~23 °C).

The SANS data reduction was performed using standard procedures to correct for detector sensitivity, instrument dark current, sample transmission and solvent background with facility-provided data reduction software MantidPlot^70^. 1-D, *I*(*q*) vs. *q*, SANS profiles were generated from the 2-D reduced data by azimuthal averaging with respect to incident direct beam.

### SANS Data Analysis and Modeling

SANS data was analyzed using a 3-layer polydisperse core-shell model. A scattering length density (SLD) profile comprising three successive shells, namely the inner headgroup (IH) adjacent to the D_2_O core inside, the hydrocarbon chains (HC), and the outer headgroup (OH) adjacent to D_2_O buffer outside vesicle, respectively. The scattering intensity from dilute vesicles using a three-shell model is given by^71^,


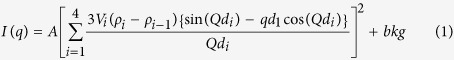


where, 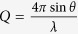, is the scattering vector for a scattering angle 2*θ. A* is the scaling factor and *bkg* is the background. *V*_*i*_, *d*_*i*_ and *ρ*_*i*_ are the volume, thickness and neutron scattering length density respectively, of each shell^65^,^72^. The subscripts, *i* = 1, 2, 3, 4 represent the core, inner head, hydrocarbon chain and outer head regime respectively. The polydispersity in ULV sizes was accounted using Schulz distribution function. The SLDs, 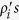 for core and solvent were fixed to that of D_2_O while the core polydispersity was fixed to 0.3. The bilayers were not constrained for symmetric SLD profile. The fitting results are averaged values from sets of converging fitting runs as previous studies^65^,^72^.

### QENS Data Collection and Reduction

QENS have been carried out on 5% (*w/w*) DMPG ULVs only and in the presence Aβ with peptide-to-lipid molar ratio 1/30 in the solid gel phase (7 °C) and the fluid phase at physiological temperature of 37 °C using the high resolution backscattering spectrometer BASIS^73^ at the Spallation Neutron Source (SNS) at Oak Ridge National Laboratory. The BASIS was used with an energy resolution of 3.4 μeV (full width at half-maximum, for the *Q*-averaged resolution value), and an energy transfer window suitable for the measurements was ±100 μeV. The samples have been placed in an annular aluminum can with an internal spacing of 0.5 mm to achieve no more than 10% scattering and thus reduce multiple scattering. Molecular motions with characteristic times faster than ~400 ps can be easily monitored with the BASIS in this set up. The quasi-elastic spectra have been recorded in the *Q* range of 0.3 Å^−1^ to 1.5 Å^−1^. QENS measurements have also been carried out on D_2_O at 7 °C and 37 °C and subtracted from those measured for the vesicle solutions. For determining the instrument resolution, QENS measurements have been carried out on vanadium. MANTID was used to carry out standard data reduction, including background subtraction and detector efficiency corrections^74^.

### QENS Data Analysis and Modeling

Two different kinds of motion, namely lateral motion of entire lipid molecules within monolayer and internal motion of lipid molecules, contribute to spectra measured at the BASIS spectrometer (ΔE ~ 3.4 μeV), based on recent results from QENS study on DMPC vesicles^35^. Therefore, *Q*-averaged scattering law for the vesicles can be expressed as^35^,


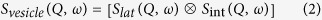


where, *S*_*lat*_(*Q*, *ω*) and *S*_*int*_(*Q*, *ω*) correspond to the scattering functions due to the lateral and internal motions of the lipid molecules, respectively. The lateral motion is well characterised by continuous flow diffusion^35^,^75^, and, therefore, the scattering law for the lateral motion can be written as,


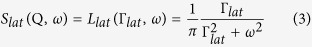


where Γ_lat_ is the half width at half maximum (HWHM) of the Lorentzian corresponding to the lateral motion of the lipid molecules. Scattering law for the internal motion, *S*_*int*_(*Q*, *ω*) can be expressed as^35^,^76^,^77^,


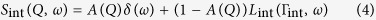


where the first term represents the elastic component and second term represents the quasi-elastic component which is approximated by a single Lorentzian function, L_int_(Γ_int,_ ω) with a half width at half maximum (HWHM), Γ_int,_. The fraction of the elastic scattering *A*(*Q*) with respect to the total scattering is known as Elastic Incoherent Structure Factor (EISF).

From equations above, the scattering law for vesicles can be written as,


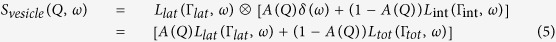


here, the Lorentzian, 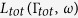 represents the combination of the lateral and internal motions of the lipid molecules, where 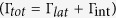. The above scattering law was convoluted with the instrumental resolution function, and the parameters *A*(*Q*), Γ_lat_ and Γ_tot_ were determined by a least squares fit of the measured spectra. The data were fitted using the DAVE developed at the NIST Center for Neutron Research^78^.

### CD, OCD, FTIR Experiment

The secondary structure of Aβ was determined using a JASCO J-810 spectropolarimeter (JASCO, Tokyo, Japan). The CD spectra were recorded on 100 μM Aβ samples with a scan speed of 100 nm/min, 0.5 s response time, and a bandwidth of 1 nm in the range of 190–260 nm. Each spectrum was an average over 5 scans. DichroWeb was used for quantitative analysis of CD data in 185–240 nm data range using SELCON tool with appropriate base set^39^,^79^,^80^.

The OCD spectra were collected from samples sealed inside a stainless steel sample cell with circular quartz windows in which a relative humidity of ~97% was maintained by a saturated potassium sulfate solution^65^,^72^. The presented OCD spectrum was an average of two OCD spectra obtained by rotating the sample by 90° around the sample normal direction in order to reduce the possible artifacts from linear dichroism.

The FTIR spectra on 100 μM Aβ (mixed with 3 mM DMPG in lipid-peptide samples) for the amide region were acquired on over the range of 400 cm^−1^–4000 cm^−1^ (of which the amide range between 1600 cm^−1^–1700 cm^−1^ is presented) with a resolution of 0.5 cm^−1^ using Jasco FT/IR-6100 Fourier transform spectrometer (JASCO, Tokyo, Japan), fitted with accessories to record attenuated total reflectance (ATR) spectra at one reflection with a diamond crystal. The spectra were measured using 100 scans at room temperature (~22 °C). The final spectra were corrected for the solvent contribution by subtraction of a reference spectrum (NaPi buffer at pH 7.4).

## Additional Information

**How to cite this article**: Rai, D. K. *et al.* Neutron Scattering Studies of the Interplay of Amyloid β Peptide(1–40) and An Anionic Lipid 1,2-dimyristoyl-sn-glycero-3-phosphoglycerol. *Sci. Rep.* **6**, 30983; doi: 10.1038/srep30983 (2016).

## Supplementary Material

Supplementary Information

## Figures and Tables

**Table 1:** Results from Core-Shell fits on SANS results from DMPG and DMPG/Aβ ULVs.

Parameter	DMPG	DMPG/Aβ
Inner Headgroup thickness, 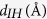	9.7 ± 0.2	9.7 ± 0.3
Inner Headgroup SLD, 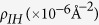	4.9 ± 0.6	4.9 ± 0.9
Hydrocarbon Chain thickness, 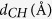	25.5 ± 0.4	25.4 ± 0.7
Hydrocarbon Chain SLD (Å^−2^), 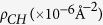	2.5 ± 0.9	2.6 ± 0.9
Outer Headgroup thickness, 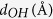	27.0 ± 1.0	20.0 ± 3.0
Outer Headgroup SLD (Å^−2^), 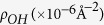	6.3 ± 0.4	6.3 ± 0.8
Total thickness, 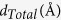	62.0 ± 2.0	55.0 ± 4.0

**Table 2:** Lateral diffusion coefficient (D_lat_) of DMPG lipid molecules in presence and absence of Aβ.

T (°C)	D_lat_ (×10^−7^ cm^2^/s)	
	DMPG	DMPG/Aβ
7	1.1 ± 0.1	0.9 ± 0.1
37	5.5 ± 0.2	6.9 ± 0.2
