# Supplementary material for: Neutron Scattering Studies of the Interplay of Amyloid β Peptide(1–40) and An Anionic Lipid 1,2-dimyristoyl-sn-glycero-3-phosphoglycerol
Source: Sci Rep. 2016 Aug 9;6:30983. doi: 10.1038/srep30983 (PMC4995599; doi:10.1038/srep30983)
Supplement: Supplementary Information [file srep30983-s1.pdf]

Neutron Scattering Studies of the Interplay of Amyloid β Peptide(1–40) and An Anionic Lipid 1,2-dimyristoyl-sn-glycero-3-phosphoglycerol

Durgesh K. Rai[1], Veerendra K. Sharma[1], Divina Anunciado[1], Hugh O'Neill[1], Eugene Mamontov [2], Volker Urban [1], William T. Heller [1], Shuo Qian [1]*

1. Biology and Soft Matter Division, Oak Ridge National Laboratory, Oak Ridge, TN 37831
2. Chemical and Engineering Materials Division, Oak Ridge National Laboratory, Oak Ridge, TN 37831

*Corresponding author: Shuo Qian qians@ornl.gov
PO BOX 2008, MS6393, Oak Ridge National Laboratory, Oak Ridge, TN 37831;
Phone: 865-241-1934
Fax: 865-574-6268

Scattering laws used for QENS analysis

Scattering used for sample below phase transition temperature:

Scattering law for uniaxial rotational model can be written as [1,2],

$$S_{\text{int}}(Q,\omega) = (1-p_x)\delta(\omega) + p_x\left[B_0(Qr)\delta(\omega) + \frac{1}{\pi}\sum_{n=1}^{N-1} B_n(Qr)\frac{1/\tau_n}{(1/\tau_n)^2+\omega^2}\right] \quad \text{(S1)}$$

with,

$$B_n(Qr) = \frac{1}{N}\sum_{p=1}^{N} j_0(2Qr\sin\frac{\pi p}{N})\cos(\frac{2\pi np}{N}) \quad \text{(S2)}$$

$$\tau_n^{-1} = 2\tau^{-1}\sin^2(n\pi/N) \quad \text{(S3)}$$

where, p_x is the fraction of mobile hydrogen atoms, N is number of sites equally distributed on a circle of radius r, j_0 is spherical Bessel function of the 0th order, and τ is the average time spent in a site between the two successive jumps. Dianoux *et al* [1] showed that for $Qr \le \pi$, the above scattering law could be sufficiently used to describe the uniaxial rotational diffusion for $N \ge 6$. In that case, the rotational diffusion constant, D_r can be written as,

$$D_r = \frac{2}{\tau}\sin^2(\frac{\pi}{N}) \quad \text{(S4)}$$

and the model elastic incoherent structure factor (EISF) can be written as,

$$B_0(Qr) = \frac{1}{N}\sum_{p=1}^{N} j_0(2Qr\sin\frac{\pi p}{N}) \quad \text{(S5)}$$

We have taken the summation for *N=12*, a large enough number (>6), which was found adequate for adopting the uniaxial rotational diffusion model for the *Q*-range used here.

After taking into account the fraction of immobile hydrogen atoms, the resultant EISF in this case would be,

$$A(Q) = p_x B_0(Qr) + (1-p_x) \quad B_0(Qr) = \frac{1}{N}\sum_{p=1}^{N} j_0(2Qr\sin\frac{\pi p}{N}) \qquad \text{(S6)}$$

Scattering law used for sample above phase transition temperature

At 37˚C, in the fluid phase acyl chains are more disordered and has large molecular area therefore are expected to undergo localized translational diffusion in which hydrogen atoms undergo localized translational diffusion within spheres whose size increase linearly from near the lipid's polar head group to the end of its hydrophobic tail, and a similar variation in the diffusivity is assumed. In the framework of this model, scattering law can be written by modifying Volino and Dianoux model, which is given by [3],

$$S_{inc}(Q,\omega) = \frac{1}{M}\sum_{i=1}^{M}\left[A_0^0(QR_i)\delta(\omega) + \frac{1}{\pi}\sum_{\{l,n\}\neq\{0,0\}} (2l+1) A_n^l(QR_i) \frac{(x_n^l)^2 D_i / R_i^2}{\left[(x_n^l)^2 D_i / R_i^2\right]^2 + \omega^2} \right] \qquad \text{(S7)}$$

The first term corresponds to the elastic component, whereas the second term is the quasielastic component, which comprises a series of Lorentzians. $A_0^0(QR_i)$ and $A_n^l(QR_i)$ $(n,l \neq 0,0)$ are the elastic and quasielastic structure factors. $A_n^l(QR)$ for different n and l can be calculated by using the values of x_n^l, R_i is the radius of the ith sphere, and can be expressed as,

$$R_i = \frac{i-1}{M-1}\left[R_{\max} - R_{\min}\right] + R_{\min} \qquad \text{(S8)}$$

D_i is the diffusivity of hydrogen atoms associated with the ith site along the alkyl chain, and can be written as,

$$D_i = \frac{i-1}{M-1}\left[D_{\max} - D_{\min}\right] + D_{\min} \tag{S9}$$

where, M is total number of CH_2 units that are taking part in the motion. For DMPG, we have taken M equal to 14. Therefore, if all the CH_2 units are taking part in the dynamics, then the averaged EISF can be written as,

$$A_{av}(Q) = \frac{1}{14}\sum_{i=1}^{14} A_0^0(QR_i) = \frac{1}{14}\sum_{i=1}^{14}\left[\frac{3j_1(QR_i)}{QR_i}\right]^2 \tag{S10}$$

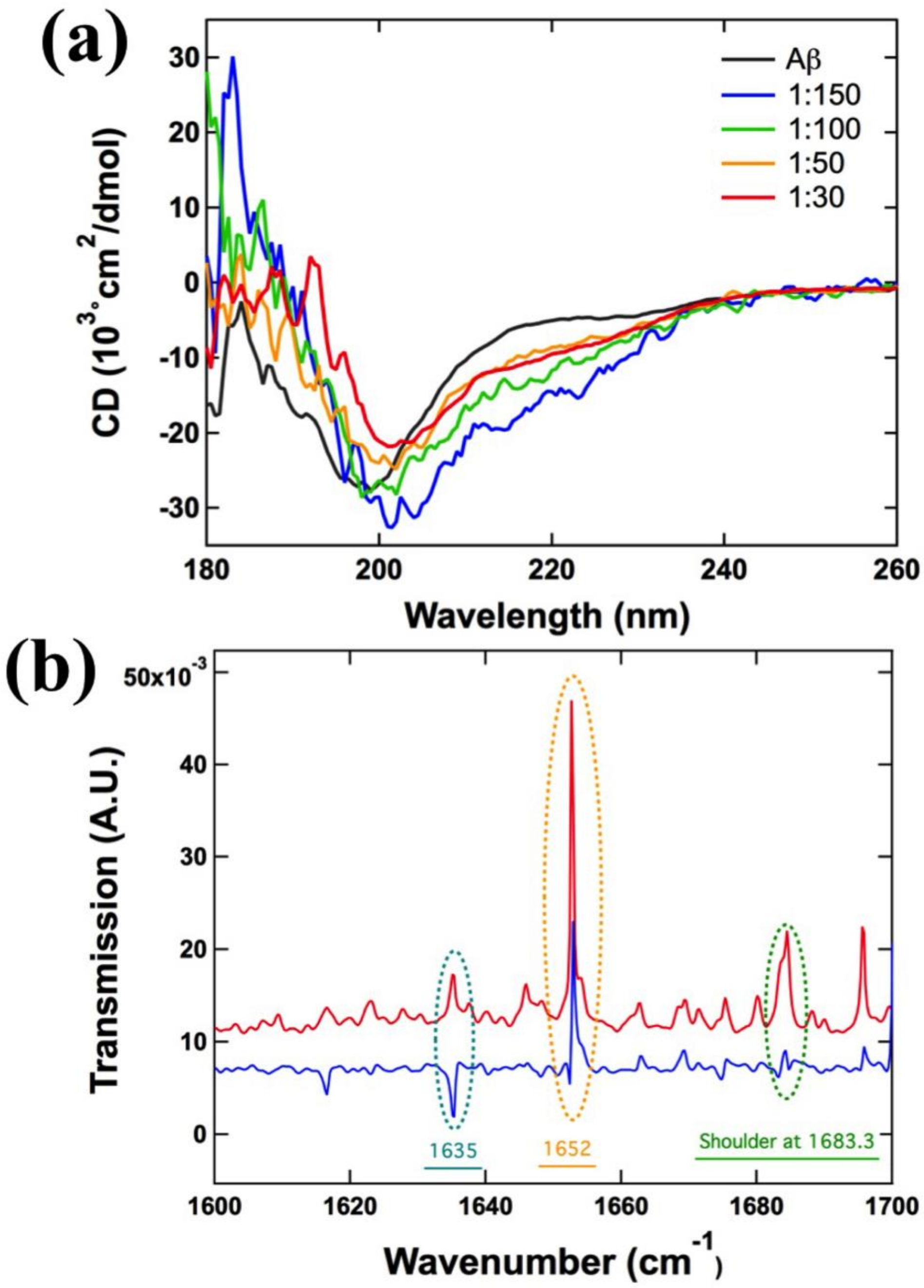

Figure S1: *(a) Molar CD of Aβ control and DMPG/Aβ (DMPG fixed at 3mM) in different rations after equilibration of 2hr at 30˚C, (b) FTIR on Aβ (red line) and Aβ (blue line) under presence of DMPG; at 100μM, 22˚C.*

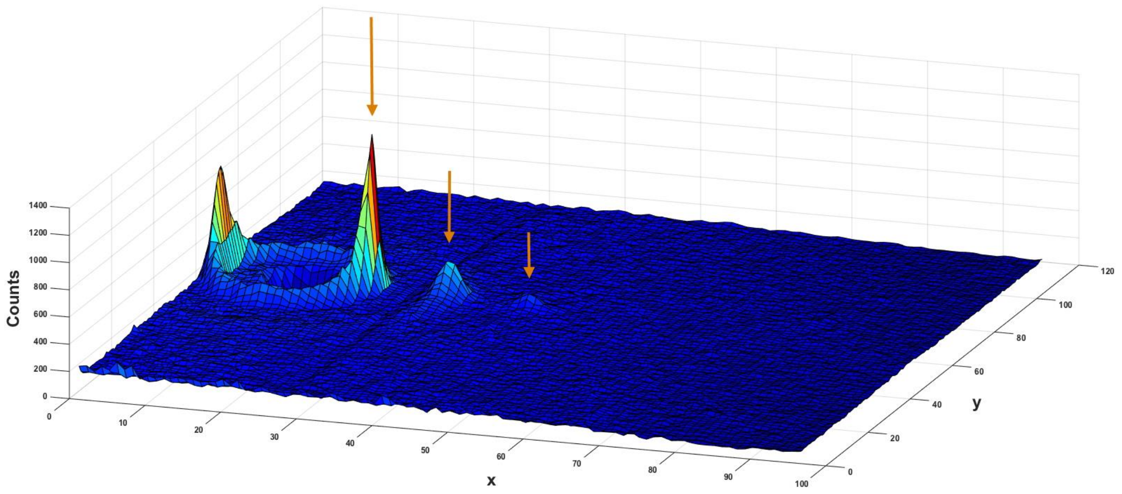

Figure S2. Typical reduced 2D Q_{xy}. *The orange circles in solid, dash and dots indicate* 1st, 2nd *and* 3rd *order peaks, which yield the NSLD profile using Swelling Method.*

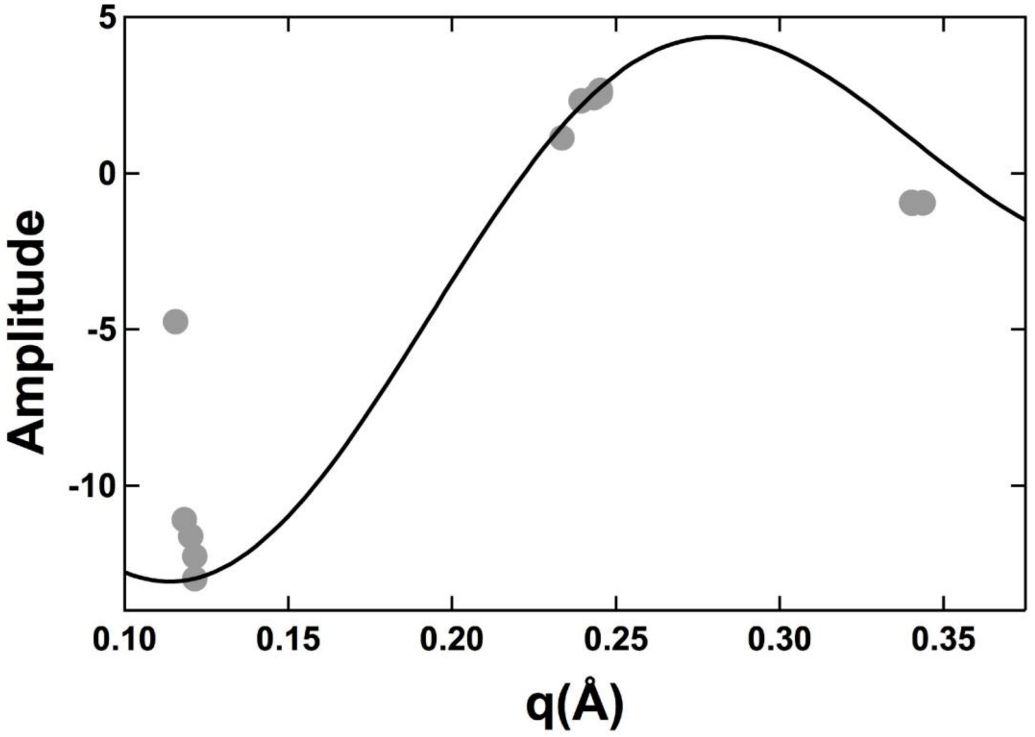

Figure S3. Phase determination for amplitudes determined by the swelling method

Table S1: Secondary structure analysis of Aβ with and without DMPG in different P/L ratios [4]

DMPG:Aβ	***Helix***	***Beta***	***Random Coil***
0:100µM	4	16	80
3mM:*100µM*	10	35	55
3mM:*60µM*	7	32	61
3mM:*30µM*	19	34	47
3mM:*20µM*	16	31	52

References

1. Dianoux, A. J., Volino, F. & Hervet, H. Incoherent scattering law for neutron quasi-elastic scattering in liquid crystals. *Molecular Physics* **30**, 1181-1194, doi:10.1080/00268977500102721 (1975).
2. Sharma, V. K., Mamontov, E., Anunciado, D. B., O'Neill, H. & Urban, V. Nanoscopic Dynamics of Phospholipid in Unilamellar Vesicles: Effect of Gel to Fluid Phase Transition. *Journal of Physical Chemistry B* **119**, 4460-4470, doi:10.1021/acs.jpcb.5b00220 (2015).
3. Volino, F. & Dianoux, A. J. Neutron incoherent scattering law for diffusion in a potential of spherical symmetry: general formalism and application to diffusion inside a sphere. *Molecular Physics* **41**, 271-279, doi:10.1080/00268978000102761 (1980).
4. Sreerama, N. & Woody, R. W. Estimation of Protein Secondary Structure from Circular Dichroism Spectra: Comparison of CONTIN, SELCON, and CDSSTR Methods with an Expanded Reference Set. *Analytical Biochemistry* **287**, 252-260, doi:http://dx.doi.org/10.1006/abio.2000.4880 (2000).
